# Woven Wearable Electronic Textiles as Self‐Powered Intelligent Tribo‐Sensors for Activity Monitoring

**DOI:** 10.1002/gch2.201900070

**Published:** 2019-11-14

**Authors:** Xiuling Zhang, Jiaona Wang, Yi Xing, Congju Li

**Affiliations:** ^1^ School of Energy and Environmental Engineering University of Science and Technology Beijing Beijing 100083 China; ^2^ School of Materials Science & Engineering Beijing Institute of Fashion Technology Beijing 100029 China; ^3^ Beijing Key Laboratory of Clothing Materials R&D and Assessment Beijing 100029 China

**Keywords:** coaxial fibers, electronic textiles, self‐powered electronics, wearable sensors

## Abstract

Wearable and shape‐adaptive electronic textiles (E‐textiles) for human activities detection such as diversity joints motion are highly desired. However, conventional E‐textiles still remain great challenges, such as flexibility, air permeability, and large‐area fabrication. Here, a fabric E‐textile is developed as a self‐powered textile for tracking active motion signals. The fiber‐shaped coaxial tribo‐sensor is fabricated with silver yarn (Ag) and polytetrafluoroethylene yarn, which allows for integrating well with cloths at large scales due to its satisfactory breathability, good washability, and desirable flexibility. Based on the coaxial‐structured design, the fabricated E‐textile is optimized to generate the output performance with maximum short‐current (*I*_sc_) of 90 nA and open‐voltage (*V*_oc_) of 8 V. Moreover, the E‐textile can also be utilized as a self‐powered activity tribo‐sensor to monitor the motion signals of the human body. More significantly, the obtained E‐textile performs outstanding finger‐touching sensitivity, which can be applied in a wireless controller, active sensor, and human–machine interactions. This work presents a new way for a multifunctional E‐textile with potential applications in smart home systems, wearable electronics, and personalized healthcare.

## Graphical abstract

The shape‐adaptive electronic textile (SET) as a self‐powered sensor generates electrical signals in response to bending and touching. The SET with good flexibility, desirable compatibility, and skin comfort performs excellent finger‐touching sensitivity to control domestic appliances wirelessly. The woven SET succeeds in real‐time monitoring of human activities for healthcare.

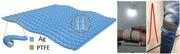

Intelligent electronic textiles, which could capture and identify diverse human motions, are drawing attention for wearable electronics^1^, ^2^, ^3^, ^4^, ^5^ due to their great potential applications in health monitoring,^6^, ^7^ deformable consumer electronics,^8^ and wearable human–machine interfaces.^9^ To date, many wearable electronics have emerged in our daily life, for example, clothes, eye glasses, wristwatch, and even smart sensors.^10^, ^11^ However, the aforementioned devices usually require batteries as a power source, which lack the desired flexibility and lightweight for devices.^12^ Furthermore, wearable electronics still remain great challenges in terms of air permeability, flexibility, and light weight.^13^, ^14^ On the other hand, smart touch‐sensing systems are crucial for human‐interactive interfaces.^15^, ^16^ Although artificial skin sensors have the advantages of high sensitivity and shape‐adaptivity, they need great improvement on skin‐friendly and comfortability.^17^, ^18^

The newly developed triboelectric devices based on the triboelectrification effect and electrostatic induction could serve as a self‐sufficient and continuous power‐supplying source.^19^, ^20^ Considering its low cost, simple structure, and universal availability, the triboelectric devices have received considerable attentions in wearable electronics.^21^, ^22^ In most reported articles, the commercial polymer films (polytetrafluoroethylene (PTFE), polyethylene terephthalate, polydimethylsiloxane) are used as flexible support which possesses low air permeability and poor skin‐friendly. Additionally, Cu foil tape and spiral steel wire are stiff and lack of desirable breathability when applied electrodes. It can be seen from the reported articles that the air permeability is mainly achieved by intercrossing their longitude and latitude lines to generate gaps, instead of the raw materials with high air permeability.^23^, ^24^, ^25^ In addition, coating conductive nanomaterials might detach from the substrates during the washing process which could hardly satisfy the requirement washability.^26^, ^27^ Moreover, the manufacturing processes (metal and polymer coating) are complicated, expensive, and time consuming.^28^, ^29^ Finally, naked metal wires and polymer films coated directly on textile substrates may cause the clothes unbreathable and uncomfortable.^30^ However, strings and fabrics can be easily integrated into cloths/textiles or directly attached on the human body for the real‐time detection of joint motions such as wrist bending, figure touch. Therefore, it is highly significant to fabricate a versatile deformable and wearable electronic textile as self‐powered smart motion sensors.

Here, a shape‐adaptive electronic textile (SET) operated as detector for human activities and a self‐powered touch sensor was fabricated for intelligent human‐interactive interfaces. PTFE and silver (Ag) yarns are sequentially wrapped around the axial metallized silver yarn through winding machine. All the materials used in this work are based on greatly soft Ag and PTFE wires, so flexible support or additional friction layer is not required. Significantly, polyvinyl alcohol (PVA) is knitted between inner dielectric layer and outer dielectric layer to enlarge the contact–separation space between PTFE and Ag and further enhance sensitivity. Considering the characteristic of preparation process, the SET can be integrated into fabrics/cloths simply at large scale. First, the fabricated SET exhibits air ultrabreathability, high sensitivity, fast response time, and stable output performance. Second, our SET could also sensitively and actively monitor the motion of human body, which endows a potential application to be used as self‐powered active motion sensors. Finally, through simply pressing a single fiber, domestic appliances such as electronic fan, light bulb, and so on can be controlled wirelessly. All these superiorities make this SET as potential applications for intelligent wearable electronics textiles and smart human–machine interactions in the near future.

To satisfy the requirements of flexibility, breathability, and softness for wearable electronic devices, the SET was fabricated in coaxial textile with core–shell and orthogonal structure for three layers, as described in **Figure** **1**a. The outer and inner layers are both silver (Ag) filaments, acting as electrodes. The medium layer is PTFE yarn as frictional layer. The knitting machine is used to weave the single thread together and to form the core–shell structure.^31^ PVA between PTFE yarns and outer Ag filaments was used to increase the contact–separation distance and ensure the output performance. Finally, the fabricated SET is further woven into wearable, multifunctional textile by needles to harvest the mechanical energy from human body motions. The cross‐sectional morphology of the SET is shown in Figure 1b‐(i). It can be seen clearly that there are some holes between the outer Ag filaments and PTFE layer, which is helpful to enhance the contact area. Moreover, the appearance of woven SET (with size of 9 × 6 cm^2^) is presented in Figure 1b‐(ii).

**Figure 1:**
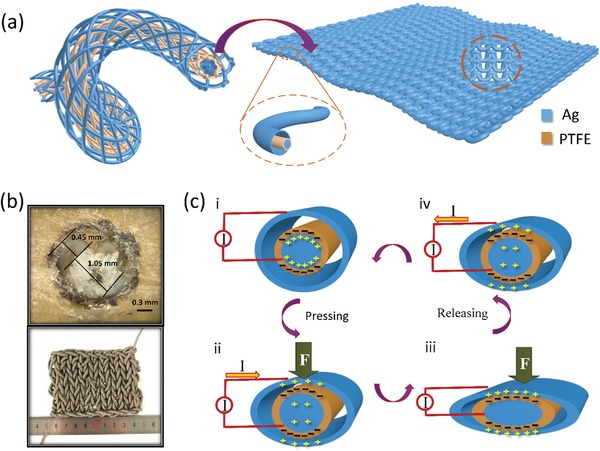
Structure illustration and working mechanism of the SET. a) The schematic illustration of the SET with coaxial structure. b) Optical microscope photograph of cross‐section and optical photograph of the SET. c) The schematic working principle of the SET.

The working mechanism of the SET is vertical contact–separation mode on the basis of the four working modes of triboelectric devices. Due to their large different tribo‐polarities between dielectric PTFE material and conductive Ag electrode, the signs of charges can be carried clearly. Figure 1c shows the charge distribution for electricity generation progress during the contact–separation processes. Once PTFE yarns contact/separate the silver filaments, the charge will transfer between friction layer and electrodes due to the potential difference. Generally, the PTFE and Ag fabrics will obtain same amount of positive and negative charges with each other after several frictional cycles. At normal state, the outer silver cannot contact with the PTFE when the pressure force is not applied on the coaxial device, then charge is neither produced nor induced between the two electrodes (Figure 1c‐(i)). Once outer Ag approaches the PTFE, electrons will flow from inner Ag electrode to outer Ag electrode to balance the potential difference between the PTFE layer and outside Ag electrode (Figure 1c‐(ii)). However, there is no current in the circuit when the outer Ag electrode is in complete contact with the PTFE, because the bound charges remains in neutralization on the surfaces of the two layers, as presented in Figure 1c‐(iii). Furthermore, when the outer Ag electrode remotes from the PTFE layer, electrons will flow back to the inner Ag electrode (Figure 1c‐(iv)). The signal generation process of the vertical contact–separation mode SET for a full cycle is described as mentioned above.

The factors affecting the output performance of SET are investigated in detail, including the tensile force when knitting the outer Ag layer and the layers of PVA in the middle of the PTFE and the outer Ag electrode. **Figure** **2**a–c shows the surface morphologies of a single compound fibers under different knitting tension for 10, 7, and 5 N, respectively. It can be found that the outer Ag yarns will become dense with the decrease of the tensile force. To measure the output performance of the SET, a linear motor was used to drive the electronic textile after being woven with the area of 9 × 6 cm^2^. Figure 2e,f shows the open‐circuit voltage (*V*_oc_) and short‐circuit current (*I*_sc_) of SET with the tension of 10, 7, and 5 N, respectively. In comparison with tensions of 10 and 7 N, the output performance (*V*_oc_ and *I*_sc_) of SET is relatively outstanding with 8.0 V and 102 nA at 5 N state. The reason can be attributed to the increasing of the contact area and conductivity of outer Ag electrode between the PTFE and outer Ag electrode. The higher density of yarns increases the contact area of friction layer and electrode, making the sufficient contact–separation, the *V*_oc_ and *I*_sc_ will be enhanced. It is necessary to point out that the layers of PVA between the outer Ag electrode and PTFE layer has a significant effect on its output performance. Therefore, different layers of the PVA yarns (Figure 2g,h) were fabricated to compare their energy‐collecting capacities. It is apparent that the output performance of the *V*_oc_ and *I*_sc_ can be gradually enhanced with the increasing number of layers of PVA. When the layers of PVA are varied from zero to three, the *V*_oc_ and *I*_sc_ increase largely from about 0.5 V, 10 nA to about 5.5 V, 80 nA, respectively, as demonstrated in Figure 2g,h. The enhanced output performance can be ascribed to the following aspects. On the one hand, the layer of PVA affects the lamellar spacing between the outer silver and the PTFE. The increasing number of the PVA layer, resulting in larger space between PTFE and Ag during the contact and separation process. On the other hand, it is demonstrated that when the radius of inner core is enhanced, the area of outer silver will be enlarged accordingly. Consequently, the increasing layers of PVA and the lower tension prompt the enhanced output performance. Therefore, the following results of SET (9 × 6 cm^2^) are discussed based on the three PVA layers and tensile force of 5 N. The output performance of SET under different resistances is also explored in this work (Figure 2d). The *I*_sc_ of the SET is almost stable before the resistance reached to 10 MΩ. Meanwhile, the peak power of SET appeared with a load resistance of 100 MΩ, which implies the matched impedance of the SET.

**Figure 2:**
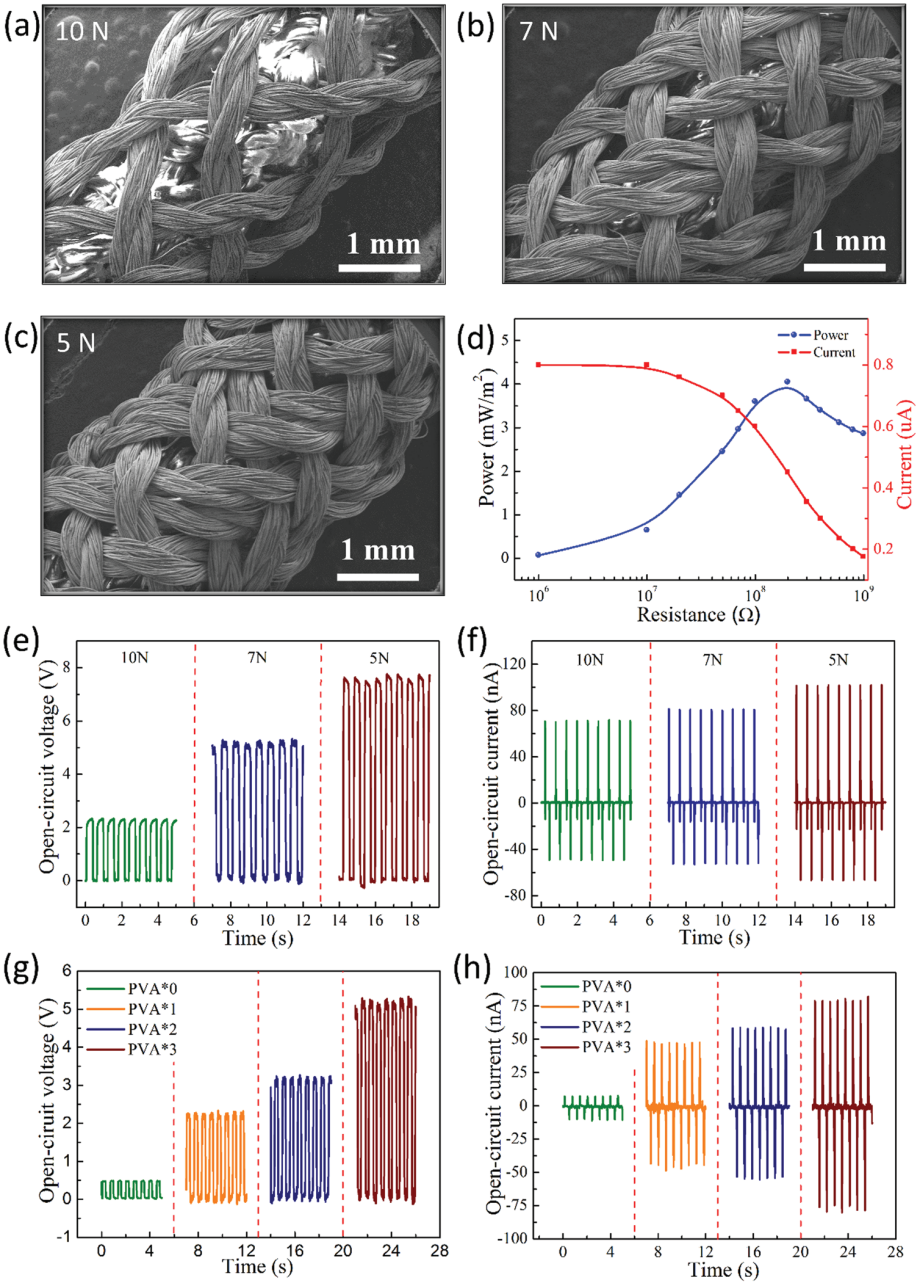
The electric output performance of the SET. a–c) SEM images of the surface of the SET under different tensions. d) Dependence of current and power on various loaded resistance. The output performances of e) *V*_oc_ and f) *I*_sc_ with different tensions. The signals of g) *V*_oc_ and h) *I*_sc_ with different layers of PVA.

Benefiting from the superior softness, flexibility, and breathability of the knitted fabrics, the SET can be applied upon various kinds of bending. **Figure** **3**a,b exhibits the *V*_oc_ and *I*_sc_ signals of the SET under various curvatures, respectively. The curvatures range from 7 to 4 cm, the corresponding optical photographs as illustrations are presented in Figure 3a,b. It is clear that the output *V*_oc_ and *I*_sc_ signs will be decreased with the increasing of the curvature, because of the higher bending ensuring the sufficient contact area between the Ag and the PTFE. Moreover, extensive applications of SET have also been demonstrated in wearable electronic devices under different deformations. Figure 3c shows the *V*_oc_ signs with peak value of about 4 V, when rubbed as the inset optical photographs. The relative video is supplied in Movie S1 in the Supporting Information. Similarly, Figure 3d–f is the *V*_oc_ signs and the according optical photographs, when muscle, elbow, and wrist twisted. It should be noteworthy that the real‐time *V*_oc_ signs of the muscle and wrist processes are not as regular as another two formations, since the two processes of contact–separation are not complete and obvious. Moreover, the *V*_oc_ sign reached to the largest value of about 5.8 V when elbow twisted, because the high strength ensures complete contact–separation and further sufficient contact area. The output performances are also measured under different manual conditions (Figure S1, Supporting Information), demonstrating its extensive applications in wearable electronic devices.

**Figure 3:**
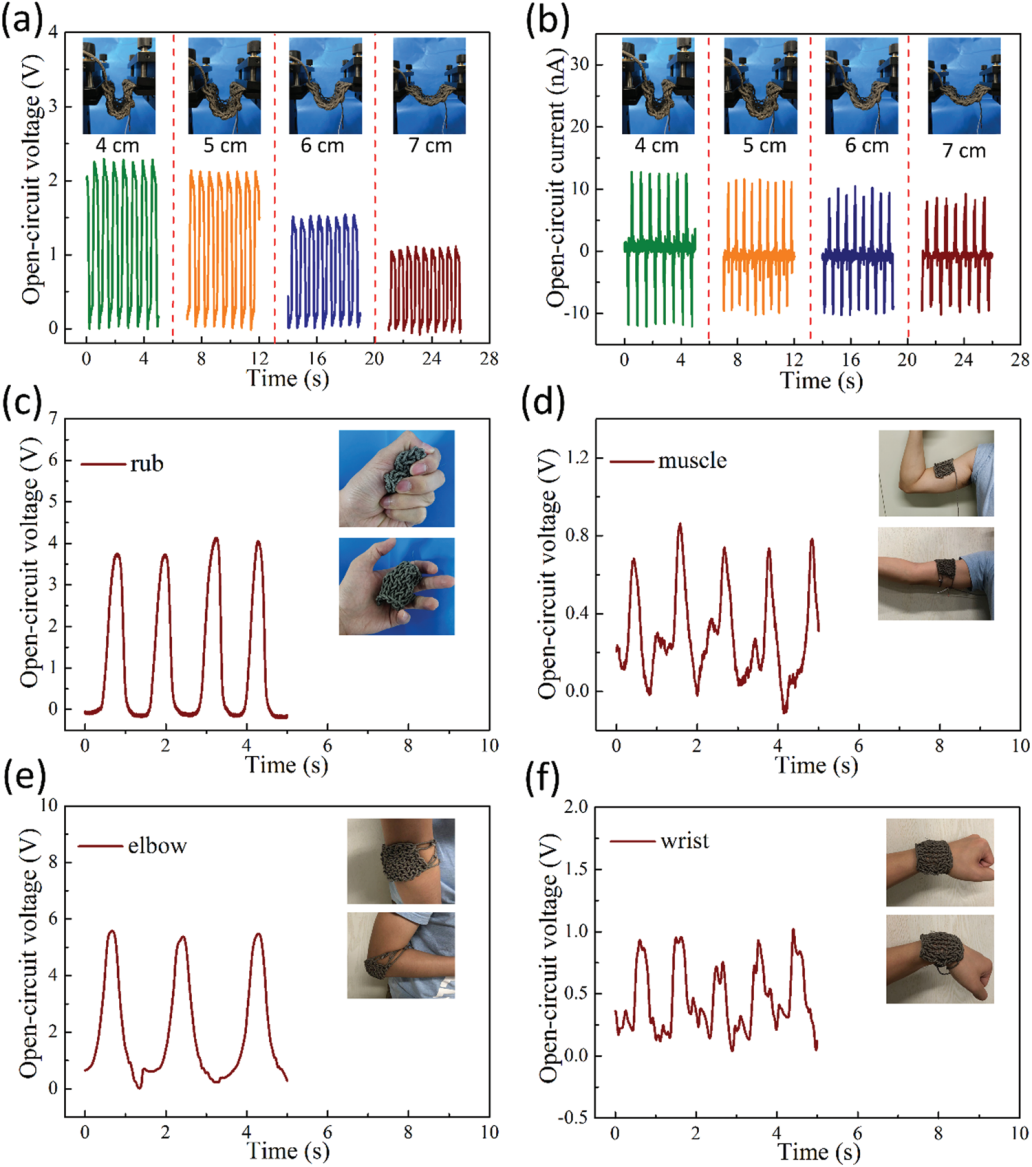
The output performance of the SET. The output of the SET with different curvatures: a) *V*_oc_; b) *I*_sc_ with curvatures ranging from 4 to 7 cm. c) The output *V*_oc_ of SET when rubbed. d–f) The applications of SET in body motion monitoring: d) muscle, e) elbow, f) wrist, respectively.

On the basis of above excellent output performance, the fiber‐shaped SET can also be used to establish a wireless smart controlling system, acting as a touch tribo‐sensor. The smart system, which consists of a single fiber, signal processing circuits, and home appliances (such as air fan and lamp), is presented in **Figure** **4**a. The fiber‐shaped SET is attached to a wristband to perceive the fingertip touch, further generating a pulse signal and trigger household appliances with the help of integrated electronic modules. The signal processing circuits are composed of four parts, and Figure 4c shows their optical photographs. Additionally, the 50‐Hz notch filter is used to eliminate the interference. Generally, the original signals generated by a single fiber were amplified and converted by AD623. Then the converted signals were sent to a latching relay which could create and transmit the converted signals to an emitter. Finally, the emitter regularly delivered the triggering signals to a receiver to control the states of an alarm, a latching relay is applied to control electrical devices like electric fan. The corresponding signals of the device from each part are presented in Figure 4b. From up to bottom, the top signal is the original of the touching signal from a single fiber, followed by filtered, amplified/converted and triggered signals. The pulse signals and electronic circuits are the key points to drive the fan.^9^, ^32^ In this process, once the SET generates a pulse signal, the relay is supposed to switch. Consequently, it is demonstrated that a lamp and electric fan can be triggered by the SET. The associated videos can be found in Movies S2 in the Supporting Information. Considering active motion and touch sensitivity of our SET, a self‐powered electronic textile can be designed in wearable electronics, which would have alternative applications in future hospitals, home security, traffic, and so forth.

**Figure 4:**
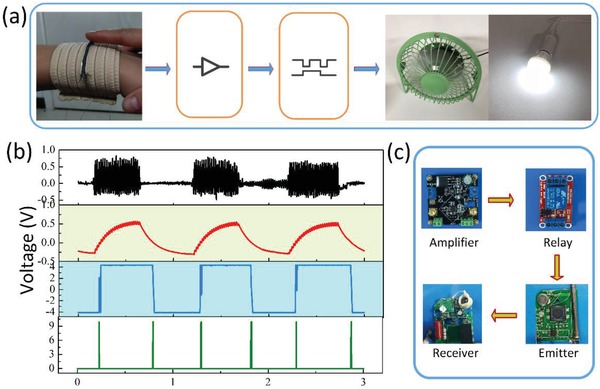
Applications of the SET in controlling home appliances. a) The scheme diagram of SET involved in smart home control system. b) The signals from top to bottom are original, filtered, amplified/converted, and triggered, respectively. c) Optical photographs of circuits used in the controlling system.

Features like skin‐friendly, cost efficiency, shape‐adaptivity, high sensitivity, and mass manufacturing of the SET also ensure its potential application in smart wearable textile to detect the consumption of calorie. The SET was attached on the knee to detect the calorie via the number of peaks of output voltage. The output *V*_oc_ at different exercise intensities are shown in **Figure** **5**a, the number of output signals is increasing when frequency enhanced. At equal exercise time, the consumption of calorie is relevant to exercise intensity and exercise time, as demonstrated in Figure 5c,d. Actually, the amount of output signals depends on the exercise frequency, further influencing the consumption energy. Therefore, it is feasible to detect the calorie according to the number of peaks, as described in Figure 5b. Due to the high durability and sensitivity, the application of the SET in smart gait recognition on the computer was further demonstrated. Figure 5e presents the signals for two persons, indicating that gait of different people can be easily recognized through our SET. To fully demonstrate the availability, the signals were also collected from another two persons (Figure S2, Supporting Information). The result indicates that there is a big difference of gait among different persons, which can be used to personal identification. Therefore, the gait recognition system might open up a new prospect to design a carpet which can achieve positioning identification indoor.

**Figure 5:**
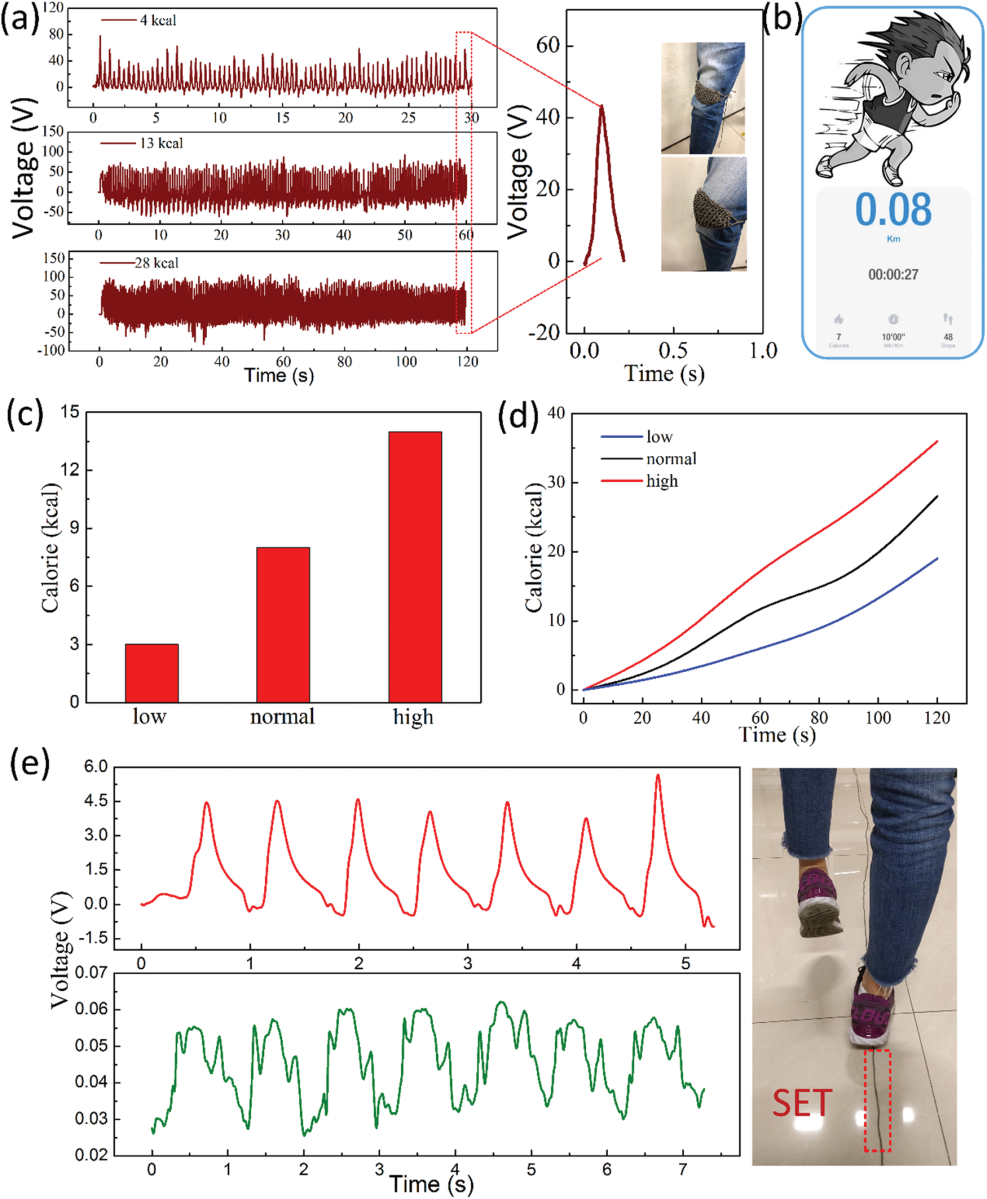
Applications of the SET in calorie consumption and gait recognition. a) The voltage output of SET when attached on the knee under different exercise intensity. b) The SET was connected to the computer to calculate the consuming calorie. c) The burned calorie in low, normal, and high frequency with fixed time. d) The change of calorie in different walking frequencies. e) The voltage signs of two people in gait recognition.

In summary, a shape‐adaptive electronic textile has been successfully fabricated with a coaxial structure. The obtained electronic textile holds advantages like high air‐permeability and excellent flexibility, which ensure its applications in wearable electronics. Moreover, the production processes of electronic textile are compatible with cloths manufacturing, indicating that it is fully capable of garment processing at large scale. Additionally, the electronic textile possesses the high output voltage and current of 8 V and 90 nA, respectively. Meanwhile, a peak power density of 4.0 mW m^−2^ at an external resistance of about 100 MΩ can be achieved. Attributed to the coaxial structure of electronic textile, the electronic textile exhibits high sensitivity and fast response towards external force. Therefore, the woven electronic textile is capable of being served as a self‐powered sensor, which can be utilized to control home appliances wirelessly such as electronic fan and light bulb. Moreover, calorie consumption and gait recognition can be also realized through the self‐powered tribo‐sensor and software on the computer. On account of the merits of being shape‐adaptive, well durable, desirably compatible, low cost, mass production, and skin‐comfortably, our fabricated electronic textile presents significant potentials in multifunctional wearable electronics, smart textiles, and human–machine interface systems in the near future.

## Experimental Section

*Fabrication of the SET*: The materials used for SET are commodity silver fiber fabric, PTFE, and PVA. The SET were fabricated through a commercial knitting machine. First, PTFE was woven around silver fiber fabric at high speed and then were twisted tightly together. Second, PVA with different layers were rotated around the above obtained PTFE layer. Third, the twisted fiber fabrics with three‐layers PVA were knitted with another silver layer under different tensile force. Finally, the preobtained was treated in water through an ultrasound treatment machine for 1 h. In order to dissolve the PVA completely, the process was repeated once. The single was successfully obtained. Then the single SET was woven together to obtain large‐area SET (9 cm × 6 cm).

*Characterization and Measurement*: The optical photograph of the compound yarns were characterized by optical microscope (LIOO IS1000). Morphologies of SET were performed on a field‐emission scanning electron microscope (FESEM, NOVA NANOSEM 450). A electrometer (Keithley 6514 system) was used to measure the short‐circuit current and open‐circuit voltage. A mechanical linear motor (Linmot, E110) was applied to drive the TENGs at an acceleration of 10 m s^−2^ and the separation distance was controlled at 10 mm.

*Signal Processing Circuit*: The initial signal of the single SET generated through touching was amplified and converted to a trigger via being linked with an amplifying circuit based on AD623 instrument. Subsequently, the signal was converted to a trigger that connected with a relay in series. After that, the relay was further associated with an emitter in series. The receiver was serially used to connect a domestic appliance.

*Intelligent Recognition System*: The designed program was based on the voltage information collected from the different persons' feet. The machine will learn from the obtained results and feedback to identify the differences between different persons. Based on the data, the gait can be recognized by the intelligent program.

## Conflict of Interest

The authors declare no conflict of interest.

## Supporting information

Supporting Information

Supplemental Movie S1
