# Supplementary material for: Woven Wearable Electronic Textiles as Self‐Powered Intelligent Tribo‐Sensors for Activity Monitoring
Source: Glob Chall. 2019 Nov 14;3(12):1900070. doi: 10.1002/gch2.201900070 (PMC6888749; doi:10.1002/gch2.201900070)
Supplement: Supplementary file 1 — Supporting Information [file GCH2-3-na-s001.pdf]

Copyright WILEY-VCH Verlag GmbH & Co. KGaA, 69469 Weinheim, Germany, 2019.

Supporting Information

for *Global Challenges,* DOI: 10.1002/gch2.201900070

Woven Wearable Electronic Textiles as Self-Powered Intelligent Tribo-Sensors for Activity Monitoring

Xiuling Zhang, Jiaona Wang, Yi Xing,* and Congju Li**

Copyright WILEY-VCH Verlag GmbH & Co. KGaA, 69469 Weinheim, Germany, 2019.

Supporting Information

Woven Wearable Electronic Textiles as Self-Powered Intelligent Tribo-Sensors for Activity Monitoring

Xiuling Zhang[a], *Jiaona Wang*[b]*, *Yi Xing*[a]*, *Congju Li*[a]*

Supporting information

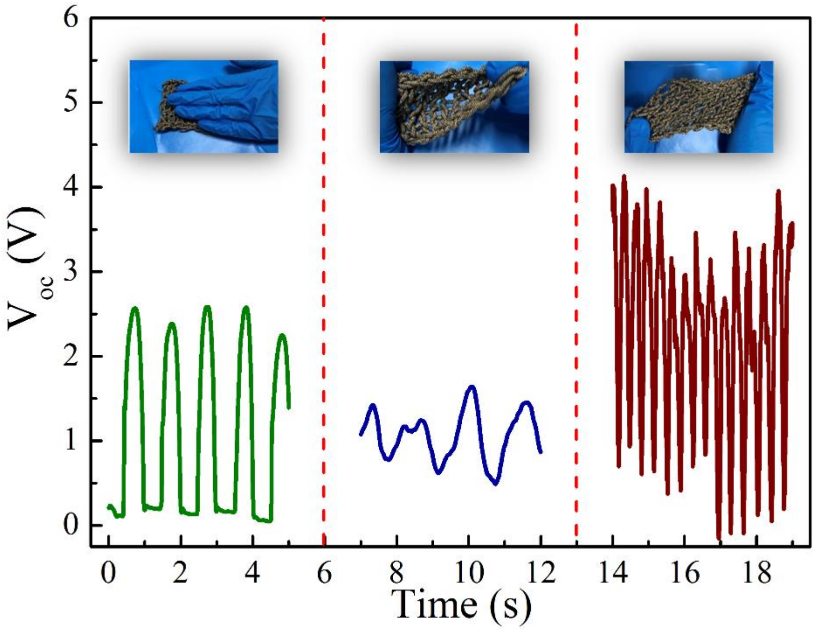

Figure S1. The V_{oc} of the SET under different bending conditions.

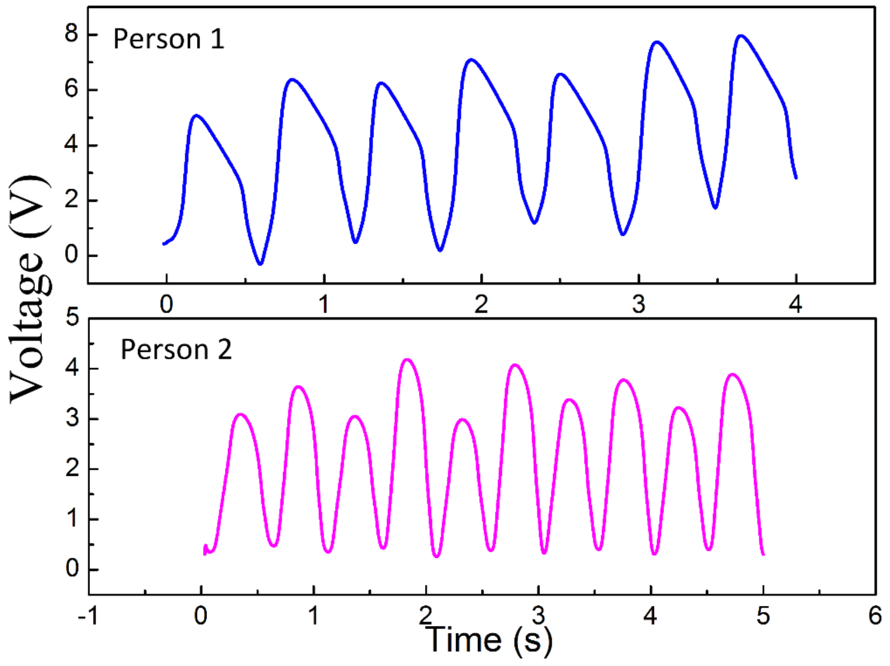

Figure S2. The voltage output of SET on gait recognition.
